# Home-based pre-surgical psychological intervention for knee osteoarthritis (HAPPiKNEES): a feasibility randomized controlled trial

**DOI:** 10.1177/0269215518755426

**Published:** 2018-02-09

**Authors:** Roshan das Nair, Jacqueline R Mhizha-Murira, Pippa Anderson, Hannah Carpenter, Simon Clarke, Sam Groves, Paul Leighton, Brigitte E Scammell, Gogem Topcu, David A Walsh, Nadina B Lincoln

**Affiliations:** 1 Division of Psychiatry and Applied Psychology, School of Medicine, University of Nottingham, Nottingham, UK; 2 Institute of Mental Health, Nottinghamshire Healthcare Trust, Nottingham, UK; 3 School of Health Sciences, University of Nottingham, Nottingham, UK; 4 Swansea Centre for Health Economics, Swansea University, Swansea, UK; 5 Division of Rehabilitation and Ageing, School of Medicine, University of Nottingham, Nottingham, UK; 6 Department of Psychology, School of Social Sciences, Nottingham Trent University, Nottingham, UK; 7 NIHR Research Design Service for the East Midlands, Faculty of Medicine and Health Sciences, University of Nottingham, Nottingham, UK; 8 Arthritis Research UK Pain Centre, Faculty of Medicine and Health Sciences, University of Nottingham, Nottingham, UK; 9 Sherwood Forest Hospitals NHS Foundation Trust, Sutton in Ashfield, UK

**Keywords:** Psychological intervention, total knee arthroplasty, knee osteoarthritis, randomized controlled trial, feasibility

## Abstract

**Objective:**

To determine the feasibility of conducting a trial of a pre-surgical psychological intervention on pain, function, and mood in people with knee osteoarthritis listed for total knee arthroplasty.

**Design:**

Multi-centre, mixed-methods feasibility randomized controlled trial of intervention plus usual care versus usual care.

**Setting:**

Participants’ homes or hospital.

**Participants:**

Patients with knee osteoarthritis listed for total knee arthroplasty and score >7 on either subscales of Hospital Anxiety and Depression Scale.

**Intervention:**

*Up-to* 10 sessions of psychological intervention (based on cognitive behavioural therapy).

**Main measures:**

Feasibility outcomes (recruitment and retention rates, acceptability of trial procedures and intervention, completion of outcome measures), and standardized questionnaires assessing pain, function, and mood at baseline, and four and six months post-randomisation.

**Results:**

Of 222 people screened, 81 did not meet inclusion criteria, 64 did not wish to participate, 26 were excluded for other reasons, and 51 were randomized. A total of 30 completed 4-month outcomes and 25 completed 6-month outcomes. Modal number of intervention sessions completed was three (range 2–8). At 6-month follow-up, mood, pain, and physical function scores were consistent with clinically important benefits from intervention, with effect sizes ranging from small (*d* = 0.005) to moderate (*d* = 0.74), and significant differences in physical function between intervention and usual care groups (*d* = 1.16). Feedback interviews suggested that participants understood the rationale for the study, found the information provided adequate, the measures comprehensive, and the intervention acceptable.

**Conclusion:**

A definitive trial is feasible, with a total sample size of 444 people. Pain is a suitable primary outcome, but best assessed 6 and 12 months post-surgery.

## Introduction

Total knee arthroplasty is an effective procedure for the management of chronic pain in late stage knee osteoarthritis.^1,2^ However, up to 20% continue to suffer pain, disability, and distress *after* surgery. Given that in 2016 there were over 100,000 knee replacement procedures conducted in the United Kingdom (the majority of which were total knee replacements^3^), with each procedure costing in excess of £7000^4^ and number of people likely to need such procedures projected to rise,^5^ the 20% who continue to suffer despite surgery represents a substantial personal and economic burden. Preoperative pain and worse mental health scores are predictive of worse postoperative pain outcomes.^6^ In particular, preoperative depression and anxiety were associated with high pain levels one to two years after total knee arthroplasty.^7,8^ Preoperative depression is also strongly associated with preoperative pain severity.^9^ Psychological distress has negative effects on functional outcomes and imposes role limitations in older patients after total knee arthroplasty.^10^

This evidence suggests that a reduction in anxiety and depression preoperatively may lead to improved postoperative outcomes. Previous studies have used ‘information giving’ preoperative classes^11–13^ to address emotional problems, but have not specifically targeted the reduction of anxiety and depression. Cognitive behavioural therapy is an effective psychological treatment for depression and anxiety and is considered to be a treatment of choice for people with these conditions.^14^ However, there is limited research evaluating the clinical and cost-effectiveness of *preoperative* cognitive behavioural therapy–based interventions to improve *postoperative* total knee arthroplasty outcomes.^15,16^

Our aim was to determine the feasibility of conducting a randomized controlled trial to investigate the clinical and cost-effectiveness of a home-administered pre-surgical psychological intervention (based on cognitive behavioural therapy) alongside usual care versus usual care alone for people on a waiting list for total knee arthroplasty for knee osteoarthritis. Specifically, we wanted to (1) assess the feasibility of recruitment and assessment procedures, (2) evaluate the acceptability of the treatment protocol and feasibility of delivering the intervention and assessments, (3) identify parameter estimates for a definitive trial, and (4) gather detailed qualitative feedback on the intervention and study procedures.

## Methods

A detailed description of the methods is published in the study protocol.^17^ In brief, this was a multi-centre, mixed-methods feasibility randomized controlled trial of a brief psychological intervention, based on cognitive behavioural therapy, plus usual care versus usual care-only control for people with knee osteoarthritis. Ethical approval was obtained from the National Research Ethics Service Committee – Nottingham 1 (reference 14/EM/0099), and the trial was prospectively registered (ISRCTN80222865).

Participants were recruited from knee surgery pathways at two UK National Health Service (NHS) hospitals. Patients attending clinic appointments were invited to complete the Hospital Anxiety and Depression Scale.^18^ Also, the orthopaedic clinical team identified potential participants from their databases and sent them an invitation letter and the Hospital Anxiety and Depression Scale.

Patients were included if they were aged over 18 years, listed for total knee arthroplasty, had osteoarthritis of the knee (defined using European League Against Rheumatism criteria),^19^ and had anxiety or depression (defined as a score of >7 on either Hospital Anxiety and Depression Scale subscale).^20^ Patients were excluded if they had co-morbid severe psychiatric conditions, had inflammatory arthritis, or were currently receiving any psychological interventions.

Eligible participants who provided written consent completed baseline assessments. These included the Western Ontario and McMaster Universities Osteoarthritis Index,^21^ Intermittent and Constant Osteoarthritis Pain scale,^22^ Beck Depression Inventory,^23^ Beck Anxiety Inventory,^24^ EQ-5D-5 L™,^25,26^ and a bespoke service-use questionnaire to assess use of NHS and social services (see online *Supplementary Document* 1).

Participants were then randomly allocated to either psychological intervention plus usual care (intervention) or usual care-only (control) on a 1:1 ratio, using a computer-generated random code, by an independent researcher not involved with the study. The recruiting researcher telephoned the independent researcher and provided the initials and date of birth of the participant, which was recorded before the allocation was revealed. The researchers and trial statistician remained blind to group allocation throughout the study. Recruitment continued until 50 participants had been randomized. This sample size was sufficient to inform the design of a phase III trial.^27^

Participants allocated to the intervention arm could receive *up to* 10 sessions of psychological intervention, based on general principles of cognitive behavioural therapy for anxiety, depression, and pain management, tailored to the needs of each participant. The intervention combined the core elements of cognitive behavioural therapy for pain management outlined by Gatchel et al.,^28^ Morley,^29^ and the Gloucester Pain Management Manual.^30^ Contents included were psychoeducation on the relationship between mood and pain, values-based goal-setting, self-management and behavioural activation, relaxation and mindful breathing, cognitive restructuring, and post-surgical planning (copies of the treatment manual are available from the authors). The hour-long sessions, scheduled to fit within the expected waiting time for surgery (maximum 18  weeks), were held once or twice weekly. One of two psychologists, trained in delivering cognitive behavioural therapy–based interventions, offered the intervention in participants’ homes or at a hospital, as preferred by the participant. To assess fidelity, therapy sessions were audio-recorded with participants’ consent.

Participants allocated to usual care did not receive any therapeutic input from the psychologists, but received the standard care delivered by each clinical service. Standard care received by the control group did not include any specific focus on the patients’ psychological state. All other clinical services were provided as usual for both groups.

Participants from both groups were assessed four and six months after randomization using the same assessments used at baseline. The outcome measures were posted to the participants with a pre-paid return envelope. Participants received assistance by telephone from a researcher if they had difficulty completing the questionnaires.

Brief semi-structured feedback interviews were conducted between follow-ups, with purposefully selected participants from the intervention (*n* = 11) and usual care groups (*n* = 12) to assess acceptability, barriers, and facilitators of the intervention and the study procedures. A maximum variation sampling strategy was used to achieve a heterogeneous sample.^31^

Treatment was coded as ‘completed’ if it was terminated by the therapist in consultation with the participant after all the key issues (goals) had been dealt with, or ‘discontinued’ if it was terminated by participants without consultation with the therapist. Descriptive statistics were used to describe the sample, indicate retention rates, and to inform power and sample size calculations for a definitive study. *T*-tests and Mann–Whitney *U*-tests (for parametric and non-parametric data, respectively) were used to compare the intervention and control groups on pain and mood outcomes. Rasch converted scores were also used, where available.^32^ Analyses were conducted on an intention-to-treat basis.

Qualitative data were analysed using a framework approach,^33–35^ which is a hierarchical, matrix-based analysis method, particularly suited where the research goals are clearly defined at the onset (e.g. to support the development of a future definitive trial).

## Results

**Figure 1.**
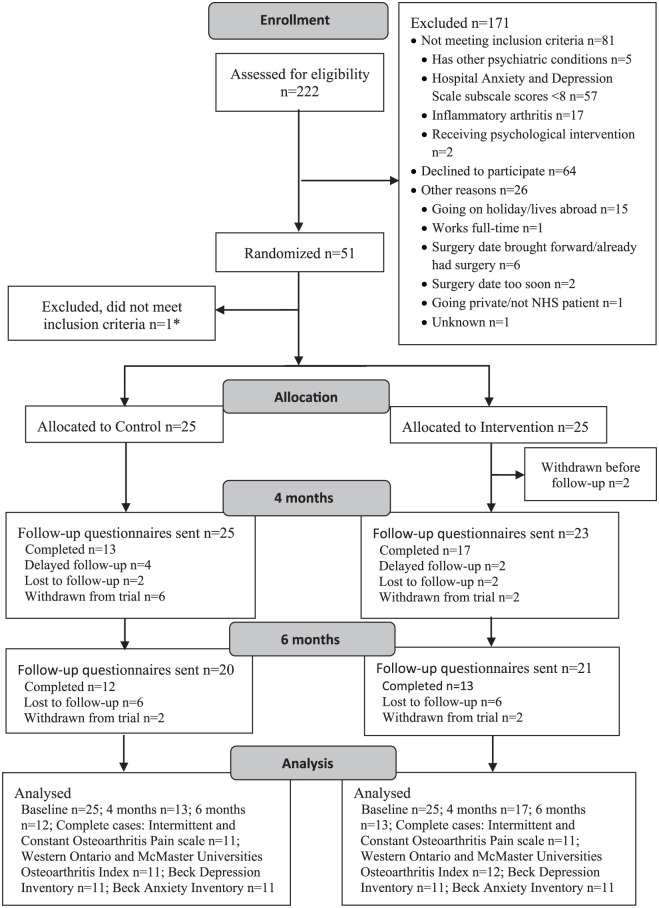
CONSORT diagram. *Included in error, due to miscalculated screening score.

In total, 51 participants were randomized, 48 from one site and 3 from the other (please see the CONSORT diagram (Figure 1)). Demographic characteristics are shown in Table 1. The groups were well-matched on demographic and surgery characteristics at baseline. The mean anxiety and depression subscale scores *for both* groups were in the ‘mild’ range (i.e. total subscale score between 8 and 10). However, using the cut-off suggested by Axford et al.,^20^ based on available Hospital Anxiety and Depression Scale screening data from 102 participants, 38 (37%) and 31 (30%) of those screened were not in the ‘normal’ range for depression and anxiety, respectively. Most scored in the ‘moderate’ range for depression (*n* = 20, 19.6%) and ‘mild’ range for anxiety (*n* = 15, 14.7%). Only a small proportion presented with ‘severe’ depression or anxiety (*n* = 5 (4.9%) and *n* = 3 (2.9%), respectively).

**Table 1.** Participant characteristics.

	Control group *n* = 25		Intervention group *n* = 25	
	*n*	%	*n*	%
Gender				
Men	16	64	11	44
Women	9	36	14	56
Occupation				
Not employed	2	8	4	16
Retired	17	68	17	68
Employed full-time	6	24	2	8
Employed part-time	0	0	2	4
Ethnicity				
White British	25	100	24	96
Black or Black British	0	0	1	4
Index of Multiple Deprivation Score (2015)				
1 (most deprived)	3	12	2	8
2	2	8	4	16
3	3	12	6	24
4	9	36	4	16
5	1	4	3	12
6	5	20	5	20
7	0	0	1	4
8 (least deprived)	2	8	0	0
Previous total knee replacement				
Yes	10	40	7	28
No	13	52	15	60
Missing	2	8	3	12
	Mean	SD	Mean	SD
Age	66.7	9.9	65.7	8.6
HADS subscale scores				
Anxiety	8.1	3.1	9.8	3.8
Depression	10.5	4.0	10.3	4.0

SD: standard deviation; HADS: Hospital Anxiety and Depression Scale.

Of the 222 participants screened, 51 (23%) were randomized. One participant was excluded after randomisation, due to a miscalculation of the Hospital Anxiety and Depression Scale baseline score to ascertain eligibility. Their data were excluded from the analyses.

At four-month follow-up, 48 outcome questionnaires were posted (two participants had withdrawn), and 30 (60%) were returned; 10 were returned with no telephone support to complete the questionnaires. At six-month follow-up, 25 (50%) of the questionnaires were returned; 19 did not require telephone support (Figure 1).

Table 2 shows the amount of missing data, and the success of obtaining these data by telephone, per scale and by data collection point. The data from the service-use questionnaire are not included here as some questions would have not been relevant for some participants and there was no ‘not applicable category’, so we were unable to tell if the data were missing or not applicable. Overall, less than 9% of data were missing at the three data collection points.

**Table 2.** Missing items and success of obtaining items by telephone follow-up.

Measure (total number of items)	Baseline				Four months				Six months			
	Items missing		Items obtained by telephone follow-up		Items missing		Items obtained by telephone follow-up		Items missing		Items obtained by telephone follow-up	
	*n*	%	*N*	%	*n*	%	*n*	%	*n*	%	*n*	%
Intermittent and Constant Osteoarthritis Pain scale	0/550	0	N/A^a^	N/A^a^	16/330	5	11/16	69	11/275	4	11/11	100
Western Ontario and McMaster Universities Osteoarthritis Index	56/1200	5	49/56	88	33/720	4.5	24/33	72	31/600	4	24/31	77
Beck Depression Inventory	4/1050	0.4	0/4	0	42/630	7	21/42	50	43/525	8	0/21	0
Beck Anxiety Inventory	4/1050	0.4	¼	25	42/630	7	21/42	50	43/525	8	0/21	0
EQ-5D-5 L™	5/300	2	2/5	40	2/180	1	0/2	0	0/150	0	0	0
*Total*	0		52		4		77		6		35	

The numerator is the total number of items missing, the denominator is the total number of items for the whole dataset at that time point (at baseline *n* = 50, at four months *n* = 30, and at six months *n* = 25).

a N/A because there were no missing data at this time point for this scale. The numerator is the amount of items that were collected over the telephone; the denominator is the total number of missing items for that scale for that time point. The percentage reflects the amount of missing data that could be obtained over the telephone.

Two participants had omitted the pages containing the Western Ontario and McMaster Universities Osteoarthritis Index items in the questionnaire booklet (120 missing items). One participant at four-month and one participant at six-month follow-up returned an empty questionnaire booklet. Most commonly missed Western Ontario and McMaster Universities Osteoarthritis Index items were those that related to use of stairs (items 2, 8, and 9), or a bath (item 20). Some participants wrote ‘no stairs’ or ‘no bath’ beside these questions. Other questions commonly missed were question 5 (pain standing upright), 13b (pain walking on a flat surface), 22 (pain getting on or off the toilet), and 23 (pain performing heavy domestic duties).

Outcome effect sizes ranged from small (*d* = 0.005) to moderate (*d* = 0.74) (Table 3). Western Ontario and McMaster Universities Osteoarthritis Index physical function scores were significantly higher in the intervention than in the usual care group six months after randomisation (*d* = 1.16).

**Table 3.** Comparison of outcomes by group allocation.

Measure	Time^d^	Control			Intervention			*p*	Cohen’s *d*
		*n*	Mean	SD	*n*	Mean	SD		
Intermittent and Constant Osteoarthritis Pain scale									
Constant pain (standard score for items 1–5)^a^	T1	13	9.5	5.6	16	9	5.6	0.83	0.08
	T2	12	6.2	3.2	13	6.2	4.4	0.99	0.005
Constant pain (standard score for items 1, 3, 4, 5)^a,b^	T1	13	7.9	4.6	16	7.0	4.4	0.62	0.19
	T2	12	5.1	3.0	13	4.8	3.7	0.82	0.09
Constant pain (converted Rasch score for items 1, 3, 4, 5)^c^	T1	13	8.5	4.7	16	7.7	4.7	0.66	0.17
	T2	12	6.0	3.2	13	5.5	4.1	0.75	0.13
Intermittent pain (standard score for items 6–11)^a^	T1	13	14.3	4.6	17	11.0	5.3	0.09	0.66
	T2	12	10.2	4.5	13	8.5	5.6	0.43	0.32
Intermittent pain (standard score for items 6, 7, 10, 11)^a,b^	T1	13	9.7	3.1	17	7.4	3.5	0.07	0.71
	T2	12	7.1	3.3	13	5.7	3.8	0.33	0.39
Intermittent pain (converted Rasch score for items 6, 7, 10, 11)^a,c^	T1	13	9.1	2.7	17	6.9	3.2	0.06	0.74
	T2	12	6.7	3.0	13	5.5	3.4	0.34	0.39
Western Ontario and McMaster Universities Osteoarthritis Index									
Pain^a^	T1	13	8.38	4.1	17	9.1	4.4	0.67	–0.16
	T2	12	7.5	2.3	13	6.5	3.6	0.40	0.35
Stiffness^a^	T1	13	4.2	2.1	17	4.29	1.5	0.84	–0.08
	T2	12	4.2	0.9	12	3.2	1.9	0.11	0.67
Physical function^a^	T1	13	32.9	15.3	17	31.3	14.9	0.77	0.11
	T2	12	32.0	4.8	13	20.9	12.7	0.009^a^	1.16
Beck Depression Inventory									
Standard total score	T1	13	12.0	7.4	16	10.3	6.9	0.57	0.24
	T2	12	11.4	9.1	12	8.3	6.5	0.43	0.40
Rasch converted score	T1	13	15.9	2.8	16	14.7	3.4	0.26	0.39
	T2	12	15.1	3.1	12	12.7	5.9	0.50	0.52
Beck Anxiety Inventory total score	T1	13	9.4	7.0	16	8.1	8.2	0.42	0.17
	T2	12	8.7	9.2	12	6.0	4.4	0.95	0.37

SD: standard deviation.

Higher mean scores indicate worse pain, functional limitations, and mood.

a Variables which were normally distributed. Normality was assumed if *Z* Skew and/or *Z* Kurtosis scores were between ± 1.96 for small sample sizes (*n* < 50) or between ± 3.29 for larger sample sizes (50 < *n* < 300).

b Following Moreton et al.^32^ – removed item 2 from Constant pain subscale and items 8 and 9 from the Intermittent Pain subscale. Raw total subscale scores were converted to an interval scale (0–16) using Rasch score values provided.

c Converted score (original units) = *m* + (*s* × logit score), where *s* = (wanted range)/(current range), *m* = (wanted minimum) – (current minimum × *s*).

d Time: *T*1 = four-month follow-up, *T*2 = six-month follow-up.

Of the 50 patients in the study, only 21 completed the EQ-5D-5 L™ at all time points (defined as complete cases). Numerically, the mean utility and Visual Analogue Scale (VAS) scores of the patients who failed to complete follow-ups 1 (T1) and 2 (T2) were lower than the complete cases, but there was considerable heterogeneity. The use of NHS resources was, in the main, equal among control and intervention groups pre-baseline, but differed between the groups at follow-ups 1 and 2. Given the feasibility nature of the trial and the small number of complete cases, no statistical testing was undertaken.

Participants received two to eight sessions of psychological intervention (mode = 3 sessions). Of the 25 participants who were allocated to the treatment group, 2 participants withdrew. One did not want to engage with any services not directly related to their surgical care. The other did not feel they would benefit from the treatment.

In total, 10 participants discontinued treatment. Three discontinued after one session because they felt they were coping well. Seven discontinued treatment after receiving more than one session, of which one participant discontinued treatment after eight sessions because they were not able to discuss the main cause of their anxiety. Seven participants did not complete treatment due to surgery being brought forward. The mean number of days between recruitment and surgery was 101.18 days (standard deviation (SD) = 58.11; range 4–277 days). Six participants completed treatment as planned. In total, 17 of the 23 participants who received the intervention consented to having their therapy sessions audio-recorded.

The overall intervention costs comprised the total staff time required to deliver the intervention, plus any travel costs incurred. The sessions were conducted by NHS Agenda for Change band 6 and 8a psychologists. The hourly pay rates range from £98 to £138 (based on 2014 Personal Social Services Research Unit^36^). The costs per patient for the intervention varied according to whether they were delivered by the Grade 6 or 8a psychologist and the time in each session. Total intervention costs (including staff time for therapy and travel and mileage costs) ranged from Grade 6 £10,148.64 to Grade 8a £15,028.24 (further data can be found in online *Supplementary Document* 2 *and* 3).

To determine the sample size for the full trial, we considered pain and mood outcomes as potential primary outcomes, that is, Western Ontario and McMaster Universities Osteoarthritis Index, Intermittent and Constant Osteoarthritis Pain scale, and Beck Depression Inventory and Beck Anxiety Inventory. Table 4 shows sample size estimates for each of these measures.

**Table 4.** Power and sample size calculations based on questionnaire descriptive statistics.

	Six months				Total sample size required^a^	Return rate		Sample size required if take into account attrition rate^b^	
	Control group *n* = 12		Intervention group *n* = 13			Control group	Intervention group	Per group	Total
	Mean	SD	Mean	SD					
Intermittent and Constant Osteoarthritis Pain scale									
Constant pain (standard score items 1–5)	6.17	3.22	6.15	4.71	1,243,664	12/20 = 60%	13/21 = 62%	1,036,387	2,072,773
Constant pain (standard score items 1, 3, 4, 5)	5.08	3.00	4.77	3.72	3560			2967	5934
Constant pain (converted Rasch score items 1, 3, 4, 5)	6.00	3.17	5.53	4.13	1874			1562	3124
Intermittent pain (standard score items 6–11)	10.17	4.49	8.54	5.56	302			252	504
Intermittent pain (standard score items 6, 7, 10, 11)	7.08	3.29	5.69	3.77	206			172	344
Intermittent pain (converted Rasch score items 6, 7, 10, 11)	6.73	2.96	5.48	3.41	206			172	344
Western Ontario and McMaster Universities Osteoarthritis Index									
Pain	7.5	2.32	6.46	3.57	266		13/21 = 62%	222	444
Stiffness	4.17	0.94	3.17	1.9	76		12/21 = 57%	67	134
Physical function	32.00	4.79	20.85	12.73	32		13/21 = 62%	27	54
Beck Depression Inventory									
Standard total score	11.42	9.11	8.25	6.52	198		12/21 = 57%	174	348
Rasch converted score	15.07	3.07	12.65	5.85	120			105	210
Beck Anxiety Inventory total score	8.67	9.16	6.00	4.34	226			198	396

a Continuous outcome test to test for superiority (intervention vs. control). Calculation based on significance level (alpha) of 5%, power (i-beta) of 80%. Mean outcome in control group, mean outcome in experimental group, standard deviation (total sample) of outcome at six months (please visit the link, https://sealedenvelope.com/power/continuous-superiority/).

b Based on higher attrition (the lower response rate between the intervention and control group–in the two columns on the left).

Framework analysis of the qualitative data highlighted three main themes, which are presented below (see online *Supplementary Document* 4 which includes a description of each theme and illustrative quotes).

The first theme encompassed participants’ experiences of being in the study. Overall, a majority of participants found the rationale of the study and the information provided clear. Some participants reported that they could not remember the finer details of the recruitment process due to the busy nature of the clinics, and feeling ‘overwhelmed’ soon after being informed that they would be receiving surgery. Most control participants understood the rationale of randomisation and did not mind not receiving the treatment. However, some control participants did not clearly understand the need for a control group.

The second theme encompassed participants’ views on the outcome measures. Participants felt the focus of the measures was good and comprehensive, asking the ‘right’ kind of questions. Some participants did not understand the connection between total knee arthroplasty and some of the questions on the generic mood and quality of life questionnaires. Furthermore, some participants objected to answering some mood questionnaire items and some found the service-use questionnaire difficult to complete. Half felt there were too many questionnaires. Although many participants were positive about the ease of completion, some participants thought some questions were contradictory or repetitive, which made them feel they had to check they were being consistent. Some participants also felt the timing of the outcomes was not right because they were still in the recovery period from the surgery at four months post-randomisation.

Finally, the third theme encompassed the treatment experiences of the participants from the intervention group. There was a generally positive assessment of the intervention, with participants expressing an understanding of the thoughts–mood–pain interaction and its relation to total knee arthroplasty. There were some initial concerns about what benefit it might offer, and in a few cases, these doubts were never lost. For these participants, pain was physical and could only be managed by medication or physiotherapy. There were some participants who did not agree with the thoughts–mood–pain interaction and reported that the efficacy of changing one’s thoughts to manage pain went only as far as the severity of pain that one was experiencing. Benefits of the intervention were described in terms of reassurance, relaxation, calmness, positive thoughts, thinking differently, and having more realistic expectations. Some participants perceived no benefit of cognitive behavioural therapy.

Where benefits of the intervention were reported, participants attributed these to the relaxation exercises, specific techniques learnt (e.g. distraction, challenging negative assessments), ‘personalising’ the therapy to their individual circumstances, psychoeducation, and signposting to relevant services. The reassurance of an expert voice was mentioned on several occasions and equally was the notion that the therapists were ‘nice’.

## Discussion

We demonstrate that despite some of the shortcoming of this study, on balance, it is feasible to conduct a definitive randomized controlled trial to evaluate the clinical and cost-effectiveness of a pre-surgical psychological intervention for those listed for total knee arthroplasty for knee osteoarthritis. To ensure the success of a phase III randomized controlled trial, some of the learning points gained from this study need to be carefully considered. Therefore, in this discussion, we outline the successes and the challenges we faced, and offer suggestions as to how to overcome these challenges.

We were able to recruit our target number of participants within the expected timeframe, but mainly from one centre. In this centre, there was a combination of clinical staff who were committed to the research project and a team of research nurses who were available to recruit participants within the clinic. Therefore, a Phase III trial will need research nurses whose main role would be to recruit participants and conduct baseline assessments. The qualitative data suggested that clinic recruitment was successful but some participants felt ‘overwhelmed’ by the trial information. While most participants understood the rationale for randomisation and the need for control groups, some did not. Other studies have also demonstrated this issue.^37,38^ Therefore, more work is needed in educating the participants about trial procedures before they are consented. Providing additional written materials (including audio–visual/multimedia presentations), additional informed consent discussions, and test/feedback techniques have shown to improve patient comprehension of study procedures.^39^

Participants received two to eight sessions of the psychological intervention within the period of being listed for total knee arthroplasty and the surgery. Not everyone who started treatment completed the intervention as planned. Indeed, of the 23 who began treatment, only 6 completed treatment. Discontinuation was due to surgery being moved forward for about a quarter of the participants, or due to personal or other reasons. The qualitative data suggested that most, but not everyone, understood the rationale of the thoughts–mood–pain interaction. This was also informally reported to the study team by the treating therapists. This may explain why some participants withdrew from the trial or discontinued treatment. The qualitative data also highlighted that therapist factors (e.g. manner, skill) might serve as a motivating factor for participants to continue with treatment. The intervention, therefore, may need to be limited to 3–4 sessions, with the therapist identifying a few key aspects to address in the sessions, to ensure that the intervention is completed before surgery.

Once randomized, the retention rate was adequate. Two withdrew soon after randomisation, eight withdrew at the four-month outcome, and a further four at the six-month outcome. A total of 30 participants (60%) completed the outcome measures at four months. At the four-month follow-up, more people in the intervention than control group completed the outcome measures on time (i.e. within two weeks of posting the outcome questionnaires), but at the six-month follow-up, the response rate was comparable. However, at six months, only 25 participants (50%) completed the outcome measures. At four months, 10 participants (20.8%) returned the questionnaires without telephone support to complete them, compared to 19 participants (47.5%) at six months. Missing items were successfully collected over the telephone. We therefore feel that support to complete questionnaires over the phone is needed, which may also improve response rates.

A key finding is that the outcome measures are consistent with clinically important benefit despite the limitations of the study. The assessment of pain, using the Western Ontario and McMaster Universities Osteoarthritis Index and the Intermittent and Constant Osteoarthritis Pain scale, was a suitable outcome. Although the Western Ontario and McMaster Universities Osteoarthritis Index physical function scores were significantly higher in the intervention than in the usual care group six months after randomisation, this is likely to be a chance finding, due to multiple comparisons. Reflecting on what can be done to improve outcome completion rates, this may be improved by using only one pain measure rather than two. This is consistent with participant feedback about the outcome measures being too many and too repetitive. We also feel that rather than using two mood measures (Beck Depression Inventory and Beck Anxiety Inventory) it may be better to use a shorter general measure of distress (e.g. General Health Questionnaire^40^).

Based on sample sizes for a definitive trial, we recommend the Western Ontario and McMaster Universities Osteoarthritis Index (pain subscale) as the primary outcome measure, for which a sample size of 133 per group is needed. Taking into account the attrition rate, the study would need to recruit and randomize 444 participants.

As a feasibility trial, outcomes were assessed only short-term (four and six months after randomisation). Some participants were confused about having to answer the same set of questionnaires twice within two months. Therefore, for a Phase III randomized controlled trial, we propose that the first outcome assessment is conducted at six months post-randomisation, when most participants would have recovered from the operation; and the second at 12 months post-randomisation, which will allow for the assessment of the longevity of the treatment effects. Another option would be to consider conducting the outcome assessments 6 and 12 months after the surgery itself. This way, if surgeries are delayed, the outcomes would be collected at a similar point of recovery from the surgery for all participants. However, if delay of surgery was not random (e.g. if the intervention contributed to delayed surgery), outcome assessments scheduled according to the date of surgery might not accurately reflect the outcome of the integrated treatment package. Irrespective of timing of outcome assessments, strategies to improve response rates of outcome questionnaires should be considered. We did not have an active control group (e.g. attention placebo group), which may have led to overestimating the intervention effects, and demand characteristics in the intervention group may have played a meaningful role in intervention–control differences. However, as this was a feasibility trial, where the objective was to test the feasibility of delivery of the intervention within a trial, it was appropriate not to have an attention placebo control group, which itself poses challenges in the randomized controlled trials of complex interventions.^41^

Our findings suggest that it is feasible to conduct a Phase III randomized controlled trial to evaluate whether providing psychological intervention while patients with knee osteoarthritis are on a waiting list for total knee arthroplasty is clinically cost-effective. Recruitment from clinics was feasible, the outcome measures were acceptable, and the post-randomisation retention rates were adequate. While the majority of the procedures used in this trial would be suitable for a Phase III randomized controlled trial, three key changes are needed. First, the research sites selected need staff dedicated to recruit participants. Second, to ensure the intervention is completed before surgery, it is limited to 3–4 sessions, with the therapist identifying which key aspects to address in the sessions. Third, outcomes are assessed at 6 and 12 months post-randomisation or following surgery, to allow for delays to surgery and for participants to recover from surgery. Furthermore, to ensure a good response rate to outcome measures, strategies such as online or telephone completion of questionnaires must be considered. These changes notwithstanding our findings suggest that a brief psychological intervention is an acceptable and feasible treatment for some participants that could improve outcomes from joint replacement surgery.

**Clinical Messages**

- Brief psychological intervention (based on cognitive behavioural therapy) is an acceptable and feasible treatment that could improve patient outcomes following knee surgery.
- A focused psychological intervention in 3–4 weekly sessions is required to permit delivery before patients have their surgery.
- Psychological intervention should be focused on the key aspects related to the individual patients’ mood.

## Supplemental Material

cre-2017-6520-File002_(1) – Supplemental material for Home-based pre-surgical psychological intervention for knee osteoarthritis (HAPPiKNEES): a feasibility randomized controlled trial Click here for additional data file. Supplemental material, cre-2017-6520-File002_(1) for Home-based pre-surgical psychological intervention for knee osteoarthritis (HAPPiKNEES): a feasibility randomized controlled trial by Roshan das Nair, Jacqueline R Mhizha-Murira, Pippa Anderson, Hannah Carpenter, Simon Clarke, Sam Groves, Paul Leighton, Brigitte E Scammell, Gogem Topcu, David A Walsh and Nadina B Lincoln in Clinical Rehabilitation
