# Supplementary material for: Home-based pre-surgical psychological intervention for knee osteoarthritis (HAPPiKNEES): a feasibility randomized controlled trial
Source: Clin Rehabil. 2018 Feb 9;32(6):777–89. doi: 10.1177/0269215518755426 (PMC5987849; doi:10.1177/0269215518755426)
Supplement: cre-2017-6520-File002_(1) – Supplemental material for Home-based pre-surgical psychological intervention for knee osteoarthritis (HAPPiKNEES): a feasibility randomized controlled trial [file cre-2017-6520-File002_(1).pdf]

Supplementary Document 1 – Service use questionnaire

In the past 3 months on how many occasions have you been to visit a health professional:

	**Because of your joint problems (enter number and reason)**	**Because of other reasons (enter number and reason)**
Example:<br>*Consulted the GP at the practice*	Number of times: _1___<br>Reasons: pain and swelling in left knee	Number of times: _2___<br>Reasons: stomach bug; and chest infection
Consulted the GP at the practice	Number of times: ______<br><br>Reasons: ___________________<br>____________________________	Number of times: ______<br><br>Reasons: ___________________<br>____________________________
Visited the Practice Nurse at the practice	Number of times: ______<br><br>Reasons: ___________________<br>____________________________	Number of times: ______<br><br>Reasons: ___________________<br>____________________________
Been to consult or visited by other health and social care professionals (e.g. physiotherapist, osteopath, occupational therapist, psychologist, podiatrist/ chiropodist, orthotist, dietician, etc.)	Number of times: ______<br><br>Reasons:<br>____________________________<br>____________________________<br><br>Which professionals did you see: ______________________<br>__________________________<br>Where did you see them?<br>_________________________<br>_________________________	Number of times: ______<br><br>Reasons: ___________________<br>____________________________<br><br>Which professionals did you see: ______________________<br>__________________________<br>Where did you see them?<br>_________________________<br>_________________________
Been a hospital in-patient	Number of times: ______<br><br>Reasons: ___________________<br>____________________________<br><br>Days spent in hospital: _______<br>Was this a medical or surgical ward? ____________	Number of times: ______<br><br>Reasons: ___________________<br>____________________________<br><br>Days spent in hospital: _______<br>Was this a medical or surgical ward? ____________

Visited hospital Outpatient Department	Number of times: ______<br><br>Who did you see there? _______________________<br>Reasons: ___________________ _____________________________	Number of times: ______<br><br>Who did you see there? _______________________<br>Reasons: ___________________ _____________________________

In the past 3 months on how many occasions have you <u>been visited at home by</u>:

	**Because of joint problems (enter number and reason)**	**Because of other reasons (enter number and reason)**
GP visited you at home	Number of times: ______<br><br>Reasons: ___________________ _____________________________	Number of times: ______<br><br>Reasons: ___________________ _____________________________
Community Nurse visited you at home	Number of times: ______<br><br>Reasons: ___________________ _____________________________	Number of times: ______<br><br>Reasons: ___________________ _____________________________
Other health and social care professionals visited you at home (e.g. physiotherapist, osteopath, occupational therapist, psychologist, podiatrist/ chiropodist, orthotist, dietician, etc.)	Number of times: ______<br><br>Reasons: ___________________ _____________________________<br><br>Which professionals did you see: ______________________ ___________________________	Number of times: ______<br><br>Reasons: ___________________ _____________________________<br><br>Which professionals did you see: ______________________ ___________________________

Are you currently on any medication for your joints or other problems? **YES / NO** (If YES, please list all medications)

1		6	
2		7	
3		8	
4		9	
5		10	

Have you have started and/or stopped in the last 3 months? **YES / NO** (If YES, please list medication)

Medication Started within the last 3 months:	*Medication stopped within the last 3 months:*

Are you suffering from any other complaints or illnesses besides your joint problems?
YES / NO (If yes, please list any illnesses and year of diagnosis)

__________________________ _____________________________

__________________________ _____________________________

What is your current employment status? (Please tick)

☐ Not employed
☐ Retired
☐ Employed full-time*
☐ Employed part-time*
☐ In Education full-time
☐ In Education part-time

*Includes self employed

Supplementary Document 2 – HAPPiKNEES Psychologist unit cost rates for patient contact (£)

NHS Bands	Hourly Rate	Minute Rate
8a	138.00	2.30
7	115.00	1.92
6	92.00	1.53

Travel costs for each trip were calculated using postcodes from hospital base to patients home, where appropriate. Travel rates from the UK government travel allowances website (see: https://www.gov.uk/government/publications/rates-and-allowances-travel-mileage-and-fuel-allowances/travel-mileage-and-fuel-rates-and-allowances) for private cars travelling under 10,000 business miles/annum (Rate: £40p/mile).

In Supplementary Document 3 below, the costs per patient for the intervention are provided according to whether they were delivered by a Grade 6, 7 or 8a psychologist.
Total intervention costs (inc. staff time for therapy + travel and mileage costs) are: Grade 8a **£15,028.24** (9988.36+4715+324.88); Grade 7 **£12,598.96** (8338.08+3936+324.88); and Grade 6 £**10,148.64** (6644.42+3179.34+324.88).

Supplementary Document 3 – Total intervention costs including staff time for delivering session, travel time and mileage costs

Pat ID	Sessions (n)	Session Duration (mins)	Travel Time/session (Rtn Journey) (mins)	Total Staff Time Costs						Mileage Costs	
				Band 8a (Hourly rate £138)		Band 7 (Hourly rate £115)		Band 6 (Hourly rate £92)		**Distance /session (Rtn Journey) (miles)	**Total Mileage Costs (£)
				Session Delivery (£)	Travel Time (£)	Session Delivery (£)	Travel Time (£)	Session Delivery (£)	Travel Time (£)		
N01	6	34.46*, 53.08*, 46.36*, 48.35*, 39.01*, 43.24*	0	610.08	0.00	509.28	0.00	405.83	0.00	0.0	0.00
N03	4	53.33, 49.01, 47.39, 44.58	44	448.50	404.80	374.40	337.92	298.35	269.28	16.4	26.24
S02	2	60.00, 60.00	26	276.00	119.60	230.40	99.84	183.60	79.56	7.0	5.60
S03	7	60.00*, 90.00*, 85.00*, 85.00*, 62.18*, 34.52*, 62.45*	0	1104.00	0.00	921.60	0.00	734.40	0.00	0.0	0.00

S05	8	58.30, 59.06, 57.31, 77.20, 58.58, 48.40, 49.31, 60.00	14	1082.73	257.60	903.84	215.04	720.25	171.36	5.0	16.00
S09	3	60.00, 60.00, 20.25	32	322.00	220.80	268.80	184.32	214.20	146.88	14.0	16.80
S11	3	60.00, 50.00, 25.52	50	312.80	345.00	261.12	288.00	208.08	229.50	22.0	26.40
S12	6	60.00, 60.00, 60.00, 53.09, 55.20, 36.56	10	748.65	138.00	624.96	115.20	498.02	91.80	3.2	7.68
S15	1	48.31	30	111.55	69.00	93.12	57.60	74.21	45.90	12.4	4.96
S18	1	30.00	28	69.00	64.40	57.60	53.76	45.90	42.84	11.2	4.48
S21	8	70.23, 47.42, 61.16, 47.37, 65.48, 64.14,	10	1056.85	184.00	882.24	153.60	703.04	122.40	3.0	9.60

		60.00, 42.11									
S22	1	20.00	24	46.00	55.20	38.40	46.08	30.60	36.72	6.6	2.64
S25	3	70.28, 54.15, 49.49	58	401.93	400.20	335.52	334.08	267.37	266.22	32.6	39.12
S26	3	52.09, 39.48, 21.14	60	261.05	414.00	217.92	345.60	173.66	275.40	24.6	29.52
S29	3	42.10, 39.45, 30.00	14	184.58	96.60	154.08	80.64	122.78	64.26	4.2	5.04
S31	6	90.00, 56.47, 61.36, 49.49, 43.25, 38.06	42	782.00	579.60	652.80	483.84	520.20	385.56	15.0	36.00
S34	5	54.00, 43.00, 49.07, 42.35, 34.27	62	513.48	713.00	428.64	595.20	341.57	474.30	25.6	51.20
S35	2	60.00, 50.13	32	253.58	147.20	211.68	122.88	168.68	97.92	11.0	8.80
S38	4	53.22*, 44.53,	30	444.48	276.00	371.04	230.40	295.67	183.60	11.6	18.56

		43.42, 51.10									
S43	2	60.00, 60.00	16	276.00	73.60	230.40	61.44	183.60	48.96	5.0	4.00
S47	1	57.02	14	131.10	32.20	109.44	26.88	87.21	21.42	4.6	1.84
S49	1	60.00	40	138.00	92.00	115.20	76.80	91.80	61.20	15.8	6.32
S48	3	60.00, 60.00, 60.00	14	414.00	32.20	345.60	26.88	275.40	64.26	3.4	4.08
Totals	**83**		**650**	**9988.36**	**4715.00**	**8338.08**	**3936.00**	**6644.42**	**3179.34**	**254.2**	**324.88**

* Sessions held at hospital – no staff travel required

** Distance between postcodes and travel time estimated via Google Maps (shortest distance without traffic) (Last accessed 14.4.16).

Supplementary Document 4. Emerging codes and categories, their descriptors and illustrative examples.

Themes	Sub-themes	Description	Illustrative quotes
Experience of being in the study	Understanding Rationale	Clarity of the study rationale	*"Well, as far as I could see, it [the study] was how […] I was going to cope with it [knee pain] mentally and physically, before and after my operation."* (Interview 4, M, face to face, Intervention group)
	Understanding research process	Clarity of the information provided<br><br>Acceptability of the recruitment process<br><br>Acceptability of the randomisation protocol	*"I thought it [participant information sheet] was very well put-together. If I couldn't understand it too much, then I'd have probably said I wouldn't have continued with it [the study] […] Like you said, you know, I don't have to take part if I didn't want to."* (Interview 4, M, face to face, intervention group)<br><br>*"And that's when they [research nurse in orthopaedic clinic] asked you to fill the questionnaire [screening questionnaire], and you didn't really know what it was about or anything […], we didn't know at first. You know, well, she [research nurse] probably explained a little bit about it, but it didn't sink in. You know, 'cause you've got other things on your mind at the time […] 'cause everything was happening. You know, all my other hospital appointments and all this. So…yeah, you know, you didn't take it all in."* (Interview 12, M, face to face, intervention group)

			"Interviewer:** If you had been randomly put into this group that didn't receive the treatment, and just received the questionnaires, how would you have felt about that?*<br>Respondent:** I don't know because I was lucky enough, wasn't I, to get the different ones? So I suppose – I still would have filled your questionnaire in […] and hope that we'd gain something from it, or you would gain something from it."* (Participant 19, F, telephone, intervention)<br><br>*"I had the pre-op, that's where the lady [research nurse] first talked about this, because she [research nurse] says 'you're a candidate for this HAPPiKNEES thing', but that's all, since then, between then and the surgery, nothing. I would have liked to have, you know, sort of, I suppose it would be a bit of reassurance and that sort of thing…"* (Participant 8, M, face to face, control)
Outcome measures	Focus of measures	Appropriate	*"Yeah, it [questionnaire] covered everything. I kept thinking some of the questions, I thought oh yeah, you know, you could relate to it."* (Interview 5, M, face to face, control)

		Not appropriate	*"[…] you know, it's a knee operation. It's not a – you've not got cancer. If it was cancer or something that was life-threatening, or something that was disfiguring, I could understand the questions [BDI and BAI] more."* (Interview 22, F, telephone, intervention group)
	Quantity	Adequate number of questions<br><br><br><br>Too many questions<br><br><br><br><br><br><br><br><br><br><br><br><br>Repetitions	*"As I say, they're quite straightforward and gave you the variety of choices."* (Interview 22, F, telephone, intervention group)<br><br>*"I think there was too many [questionnaires] personally and I think that would probably put a lot of people off […] especially somebody on their own, with two of us, we looked at it and went through it together, but I can imagine if somebody was on their own looking at that they'd 'oh', you know, 'I can't be bothered' […] I think people will fill forms in, the least they are the better […] I think people just get fed up with filling forms in. Whereas if it's just a quick couple of pages, I don't think people will mind so much"* (Interview 8, M, face to face, control group)<br><br>*"They asked the right questions but they were asking about – one question about four different ways. You know what I mean? […] Which I think was a bit balmy."* (Interview 15, M, telephone, control group)

	Quality	Positive views (ease of completion and understanding)<br><br>Negative views (contradictory, duplications, difficulty in understanding questionnaires)	*"Yeah, they [questionnaires] were fine. Yeah, quite easy to fill in. No problems. Straightforward"* (Interview 1, F, face to face, control group)<br><br>*"The forms [questionnaires, yeah. I'm almost certain them forms were too many […] I've never been any good with at school like I was saying and these questions they're bloody hard some of them. I can't, I'm not very good at spelling. I'll be truthful…"* (Interview 17, M, telephone, intervention group)
	Timing of the questionnaires	Concerns about the timing when they received the questionnaire (soon after surgery)	*"I found it [timing of the questionnaires] all right, but I don't know if I filled that [questionnaire] in 'correctly', because if I'd have filled that in before I'd had my operation, the answers to my questions might have been different […] But because I've had my knee done and I'm in that much pain, and it's saying to you 'in the last so many weeks, how have you felt?' […] Because of my negative thoughts, you see, because of – how the question's worded and how you've got to answer, and I try to answer as truthfully as I can because over the past through weeks, how has it been? But it was a few weeks after I'd had the operation, so you got the moany one, whereas you might have got a better one before I'd had it done."* (Interview 19, M, telephone, intervention group)

		Confusion about filling in the questionnaires at follow-ups	*"And then I when I received your letter about the pain […] I didn't realise it [questionnaire] was post-op. You know what I mean? […] I sent it [questionnaire] back. I'd already had the surgery […] I thought, well, I've had the surgery now. Why do they want…? You know […] And I thought, 'Oh, they've probably overlooked it and sent it [questionnaire] me again. Maybe they lost the other one or something.' […] did it [cover of questionnaire] say post-surgery on it, or not? If you'd highlighted that a little bit more"* (Interview 14, M, face to face, intervention group)
	Specific comments on specific outcome measures	ICOAP (confusion over the constant and intermittent pain subscales)<br><br>Mood questionnaires (connection unclear between TKR and mood questionnaires)	*"Yeah I did find some of it [questions] a bit strange cos you see, I mean the first one 'How intense is your constant knee pain?' and I tried to separate constant because I didn't get really intense constant, I mean I suppose everybody's different aren't they so what's going to be relevant to some isn't to others. Mine's more of like a nagging, always there, yeah. The constant knee pain threw me a bit cos it, it comes and goes and it can depend on what you've done the day before or I sometimes wonder what I've eaten or yeah." (Interview 6, F, face to face, intervention group)*

		EQ-5D-5L (unclear whether EQ-5D-5L questions were specific to OA or general health, confusion over too many options)	*"Some of these questions I found a bit strange like agitation and loss of interest, you know, I find those a bit, I suppose some people might do, like worthlessness, I do not feel I am worthless, you know, questions like that, I mean, I don't know if, I just find those a bit strange, them [sic] questions. I suppose some people might but why would having knee replacement make you feel worthless? I don't know. Well, I mean, I did try to answer everything as much as I can […] It seemed like this, feelings of choking, you know, what's that got to do with a knee replacement!"* (Interview 8, M, face to face, control group)<br><br>*"So I was itching, all these hives and they were like, I'd got them in my ears didn't I? And I sat and I thought well I'm going to get this out the way but how am I focusing on these when I've got all this. And then I think there was one bit in the survey where it said 'And how do you rate yourself today?' or something […] At the end, is it the one with the ruler, on a scale of zero to a hundred or something, how good is your health today […] And I thought well that, cos I thought, and this survey it was really difficult to do because I'd got extra problems other than – is this my survey or is this another one?"* (Interview 6, F, face to face, intervention group)

		Service use questionnaire	*"Some of the questions – like, you've got that many options; it confuses you a little bit. It [questionnaire instructions] does say don't think about it too long, doesn't it? Like, you know, try and read them and give your answer […] It was it on the last page, where you've got that chart [EQ-5D-5L visual analogue scale] thing? […] Yeah, I think probably sometimes you've answered one question and you go onto one probably two or three bits later on and I think, 'Well, am I going to contradict myself here, or what?' You know what I mean?"* (Interview 14, M, face to face, intervention group)<br><br>*"I think perhaps I felt that there wasn't quite enough space [on questionnaire]. But I've been back for several other things and there wasn't quite enough room there [on questionnaire]."* (Interview 6, F, face to face, intervention group)
Treatment experience	Rationale (understanding and acceptance of CBT)	Understanding of the relationship between thoughts, mood and pain	*"Well, I guess my thought process was how I dealt with pain. That to me was enlightening. Then when I went to the talking session, I found that useful with we sort of talked about relaxation and anxieties and questions that may have come up. So that was useful and I think that's something that I've kept with me when I've been in pain about being more*

		Misunderstandings about when the CBT should/did occur (perception that therapy should have been provided after surgery)<br><br>Understanding of CBT (CBT as a common sense, e.g. having a bit of a chat)<br><br>Concerns about what benefit it might offer	*relaxed."* (Interview 13, F, telephone, intervention group)<br><br>*"And then a nurse [research nurse] came and took all my details then and then [therapist] came pretty quickly after, probably only just over a week, I think it was less than a fortnight I went on the programme, so I suppose I was a bit early really, cos obviously there was no operation date mentioned and I'd have finished the programme well before the operation. And I did say to [therapist] at the time when I did it, I felt it [therapy] would have been more useful afterwards, after the operation but obviously you don't do that at the moment."* (Interview 6, F, face to face, intervention group)<br><br>*"[…] 'cause it's – that's nice, when somebody will come out and have a chat with you about things like this [referring to the content of the CBT sessions]. I think that's very, very good. […] Instead of just going into it [surgery].That does actually help. It [session with therapist] gives you a bit of…how can I put it? It gives you a bit of gall. It mean – what I'm trying to say is that chat helped me to go and actually get it [surgery] done"* (Interview 20, F, face to face, intervention group)

		Disagreement with the thoughts-mood-pain interaction	*"I found it [therapy sessions] a bit deep if anything because I don't see how you can manage pain when you don't know what is going to happen. I mean it's alright saying you can manage pain, but you can't manage pain unless you've got some form of medication."* (Interview 10, M, telephone, intervention group)<br><br>*"[…] to me, the pain I've suffered with my knees, I find it difficult to accept that a lot of it's in your mind, sort of thing, like – you know. […] It [therapy] was saying 'mind over matter'. Your brain sends a signal and it's the brain telling you that you're in pain and all that sort of thing. And I can understand that, but it don't help you when you're in pain in my opinion, like, anyway. And a lot of it can be mind over matter, but it depends how much pain you've got."* (Interview 14, M, face to face, intervention group)
	Perceived benefits of CBT	Example benefits: Reassurance, relaxation, calmness, positive thoughts, thinking differently, having more realistic expectations	*"Sometimes I'm finding myself walking and I'm really tense so I say to myself 'relax, relax' and I've found that that helps. So I guess that's something that I've taken away with me from doing one of the sessions […] Right, OK. Well, I guess my thought process was how I dealt with pain. That to me was enlightening. Then when I went to the talking session, I found that useful with we sort of*

			talked about relaxation and anxieties and questions that may have come up. So that was useful and I think that's something that I've kept with me when I've been in pain about being more relaxed." (Interview 13, F, telephone, intervention group)
		No perceived benefit of CBT	*"I thought it [CBT] was different to what it is […] I don't know. […] I've had two sessions of physiotherapy – by that I mean I've met the physiotherapist and she's taught me different exercises, and I've found that more helpful […] Yeah, I found that more useful – more – even though, when I do the exercises, I do know that I've done them, but I've found that is – at least I know I'm getting somewhere, or I think I'm getting somewhere."* (Interview 22, F, telephone, intervention group)
		Need for post-surgery reminders to help remember what they learned during the sessions	*"What about a tape or something that you could just put on and think, 'Oh, I'll just refresh my mind and everything with that'? That's only a thought. That's only me. […] Yeah, when I last saw [therapist], I definitely – I'm not saying everything worked 'cause, I mean, we're all individuals, but I did start and think of more positive things, and I did, when I went to the hospital, think, 'Now—' you know, 'cause it's a little bit daunting. It's early in the morning, blah, blah, and I did think, 'Now,*

			don't forget what you've been told. Relax and….' And I did. You know. But since I've been home and I'd – there aren't been many positive thoughts. I have thought, occasionally, when it's been a bit easier, 'Oh, well, you're on the mend', but then I've had a set-back and the good thoughts get pushed really, really back. But if we've got something to jog our memories. You know: don't be negative; be positive." (Interview 19, M, telephone, intervention group)
	Perceived mediators of change	CBT-specific input (e.g., relaxation and distraction techniques)<br><br>General therapy/therapist factors (e.g., personalisation of therapy, reassurance of an expert voice)	*"I mean for me the main thing [benefit of therapy] was the relaxation and having a positive outlook […] The relaxation […] was about centring [sic] on your breathing and not tensing, especially when walking, not hunching your shoulders and it talked about being in a darkened room and moving through and centring on your breathing and releasing the pain from your body."* (Interview 13, F, telephone, intervention group)<br><br>*"No it was all helpful. But saying that it may be because looking back I perhaps steered [therapist] to what was happening to me at that time. So [therapist] gave me advice personal to me in a way that was like the time management and pacing and breaking things down, to do things when, the physical stuff perhaps when the pain was less and then do*

		Other factors (e.g. information provision, sign-posting)	*my crafts when it was quite painful because it would wear off if I'm doing something else, obviously, so not to have too much of a set pattern was useful, just different ways of looking at how I was doing things."* (Interview 6, F, face to face, intervention group)<br><br>*"I enjoyed having the one to one sessions. […] it was a good way to prepare and get a mindset of having a big operation. And I felt there was a little bit of extra support there as well, apart from obviously just having the consultant, but I felt by going to the sessions if there was something I was worried about, somebody would say 'oh, you can ask the physio'. It was nice having that little bit of extra support."* (Interview 13, F, telephone, intervention group)
	Format of treatment sessions	Home or hospital setting (home for pragmatic reasons [time, transport and mobility issues] & hospital for work obligations [easier to attend during the day])<br><br>Group vs. individual treatment delivery	*"I would have gone to the hospital, but it's just, well, driving – you're not too keen on driving on motorways and dual carriageways, so it's better at home, for me."* (Interview 12, M, face to face, intervention group)<br><br>*"Possibly but I think sometimes you can get intimidated, if you know what I mean, some people kind of demand the stage, if you know what I mean […] And then other people who need to ask questions don't because they're frightened of being thought to be stupid or*

			something, you know. So I think it really depends and obviously you will always get a mixture of patients so you're going to get this mixture of somebody who's really bombastic and then somebody who's very timid." (Interview 9, F, face to face, intervention)<br><br>*"I probably think it [therapy] would be better in a group […] To be honest because I think, like I say, different people have got a different way of interpreting things and I think as a group you could probably talk about it and get more out of it that way."* (Interview 10, M, telephone, intervention group)

Note. Quotes are followed by a description of interview number, gender of the participant (M or F), whether the interview was conducted face-to-face or over the telephone, and whether the participant was allocated to the intervention or control group.
